# The Association Between Patient-Ventilator Asynchrony and Clinical Outcomes in Mechanically Ventilated Patients: A Systematic Review

**DOI:** 10.1097/CCM.0000000000006816

**Published:** 2025-08-12

**Authors:** Melissa J. de Bie, Petra J. Rietveld, Franciska van der Velde-Quist, Nan van Geloven, Jacob W. M. Snoep, Evert de Jonge, Abraham Schoe

**Affiliations:** 1 Department of Biomechanical Engineering, TU Delft, The Netherlands.; 2 Department of Intensive Care, Leiden University Medical Centre, Leiden, The Netherlands.; 3 Department of Biomedical Data Sciences, Leiden University Medical Center, Leiden, The Netherlands.

**Keywords:** artificial respiration, critical care, patient outcome assessment, patient-ventilator asynchrony, systematic review (publication type)

## Abstract

**OBJECTIVES:**

To evaluate associations between patient-ventilator asynchrony (PVA) and clinical outcome measures.

**DATA SOURCES:**

For this systematic review, the databases of PubMed, Web of Science, Embase, Cochrane Library, and Emcare were screened until June 20, 2024.

**STUDY SELECTION:**

The main inclusion criterion was the assessment of the association of PVA with clinical outcome measures (length of ICU stay, mechanical ventilation duration, and mortality).

**DATA EXTRACTION:**

All forms of PVA subtypes reported in the articles were systematically collected. Furthermore, the method used to identify asynchrony and the clinical outcomes described were recorded from each study.

**DATA SYNTHESIS:**

A total of 19 studies were included with a total of 2672 patients. The results of the meta-analysis show that overall PVA and ineffective triggering and double triggering are associated with a longer duration of mechanical ventilation (mean difference, 3.29 d; 95% CI, 0.13–6.44 d), and with a longer ICU length of stay (mean difference, 3.65 d; 95% CI, 1.20–6.11 d). No association was found between PVA and mortality. In addition, reverse triggering appears to have a potential positive association with outcome.

**CONCLUSIONS:**

PVA and specifically ineffective triggering and double triggering, are associated with a longer duration of mechanical ventilation and longer ICU length of stay.

**KEY POINTS**

**Question**: Is there an association between patient-ventilator asynchrony and clinical outcome measures?

**Findings**: This systematic review investigates the association between patient-ventilator asynchrony and clinical outcome measures. Patient-ventilator asynchrony is significant associated with a prolonged mechanical ventilation and ICU length of stay.

**Meaning**: The occurrence of patient-ventilator asynchrony can have a negative effect on outcome and should possibly be avoided.

In the ICU, complex care is provided to critically ill patients. Many of these patients are mechanically ventilated. When there is a lack of synchronization between the ventilator assistance and the patient’s breathing patterns, we speak of patient-ventilator asynchrony (PVA) (1). PVA is a common problem encountered in the management of patients receiving mechanical ventilation (MV) (2–4). There are different types of PVAs occurring during the respiratory cycles (5). Currently, there is no widely accepted gold standard for detecting PVA. The most commonly used method for PVA detection is according to visualization of flow, pressure, and volume waveforms, but this approach has limited accuracy (6, 7). Therefore, additional signals such as esophageal pressure and electrical activity of the diaphragm can be used to provide more detailed information and improve detection (8, 9).

Asynchrony can lead to a higher work of breathing, which leads to excessive strain on the respiratory muscles. This, in turn, can lead to more dyspnea and discomfort (3, 10).

Several studies have been published describing the effect of PVA on clinical outcomes, but the results are inconsistent. One review by Kyo et al (11) examined the effect of asynchrony in general and ineffective triggering in particular on the duration of MV, ICU and hospital mortality, reintubation, and tracheostomy placements. Their results indicated that patients with more than 10% asynchrony are at risk of prolonged MV and increased ICU and hospital mortality (11). However, the certainty of evidence for these results is low, and the study dates from 2021.

Furthermore, there are studies, such as those by Blanch et al (12) and Ge et al (13), that show inconsistent results, particularly regarding mortality. Additionally, Kyo et al (11) did not examine different types of asynchronies separately, which recent literature suggest is significant.

Therefore, our review aims to address these gaps by examining the effects of each type of asynchrony separately. Also, the most recent articles will be included to provide an updated and comprehensive analysis. To achieve this, systematic review with meta-analysis is conducted to investigate if there is an association between PVA and clinical outcomes in mechanically ventilated patients in the ICU. This approach will help to clarify the specific impacts of different types of asynchrony on clinical outcomes, potentially offering more precise guidance for clinical practice.

## MATERIALS AND METHODS

The Preferred Reporting Items for Systematic Reviews and Meta-Analyses 2020 guidelines were followed and can be found in **Appendix A** (https://links.lww.com/CCM/H776) (14).

### Inclusion and Exclusion Criteria

Studies were included if the effect of the occurrence of PVA on at least one clinical outcome measure was described in mechanically ventilated patients.

Studies were excluded if they involved noninvasive ventilation, and studies performed on neonatal, pediatric, and brain-injured patients were excluded. The search was limited to English articles. In addition, reviews, editorials, and case-studies were excluded.

### Outcomes of Interest

The outcome measures were mortality both in the hospital and the ICU, duration of MV, and ICU length of stay. Weaning failure was considered as an extra outcome measure.

### Definitions

The different types of PVA were defined as follows.

Ineffective triggering is defined as failure to recognize a patient’s breathing attempt by the ventilator. Double triggering means that the ventilator delivers two breaths in response to one effort inspiration of the patient. This occurs when the duration of initial support provided by the ventilator is insufficient, leading to the occurrence of two triggers (15, 16). Short cycling or early cycling occurs when the ventilator ends flow delivery, but the patient’s inspiratory effort continues (5). Prolonged cycling or delayed cycling is defined as mechanical insufflation that continues after inspiration has ceased or even during expiration. With auto-triggering, the ventilator triggers an unscheduled breath that was not initiated by the patient (17). In reverse triggering, a first breath is initiated by the ventilator and the second by the patient. This usually occurs in patients on controlled ventilation and in sedated patients. The occurrence of reverse triggering can lead to breath stacking (18). Flow starvation is defined as inadequate gas delivery, this occurs when the ventilator flow is too low for the patient’s needs. This can lead to excessive muscle strain, this is most often seen in volume assist-controlled ventilation due to the fact that flow is controlled (5, 19, 20).

The asynchrony index (AI) is a common metric used in many studies to quantify the degree of asynchrony. A threshold of 10% has been commonly adopted to define significant asynchrony, based on its association with clinical outcomes (16).

### Search Strategy

The full search strategies can be found in **Appendix L** (https://links.lww.com/CCM/H776). In short, PubMed, Web of Science, Embase, Cochrane Library, and Emcare were searched until June 20, 2024, for articles meeting the inclusion criteria.

Three authors (F.v.d.V.-Q., P.J.R., M.J.d.B.) independently reviewed the articles on eligibility by title and abstract, and those titles and abstracts that did not agree among all three reviewers were reviewed and decided together. The remaining articles that were suitable based on title and abstract were then independently assessed for full text for inclusion. If there was no agreement, discussions were held with the three reviewers until agreement was reached. The decision for inclusion of the articles used in this review was unanimous.

### Data Extraction

During the data extraction process, all forms of PVA subtypes reported in the articles were systematically collected. Furthermore, the method used to identify asynchrony and the clinical outcomes described were recorded from each study. Clinical variables relevant to the analysis, including method of detecting asynchrony, use of sedation, and comorbidities, particularly chronic obstructive pulmonary disease (COPD) and acute respiratory distress syndrome (ARDS), as well as ventilation mode, were extracted from the articles. Ineffective triggering, double triggering, and reverse triggering and their effect on outcome measures were described separately.

### Quality Assessment

The articles were independently assessed for quality by two authors (M.J.d.B., P.J.R.) using the Quality in Prognostic Studies (QUIPS) tool, which assesses the risk of bias (21). Any conflicts were resolved through discussion. The QUIPS tool scores the risk of bias on multiple components, including Study Participation, Study Attrition, Prognostic Factor Measurement, Outcome Measurement, Study Confounding, and Statistical Analysis and Reporting to get an overview of a particular study’s risk of bias.

### Statistical Analysis

Statistical analyses were performed with R, Version 4.2.1 (R Foundation for Statistical Computing, Vienna, Austria) in RStudio, version 2022.02.3 + 492 (Posit, PBC, Boston, MA). Meta-analysis were performed of the outcome measures as described above, including a meta-analysis with the studies that measured asynchrony on multiple days.

The meta-analyses were performed with a random effect model. The effect size for continuous outcome variables is expressed in mean difference (MD) with a 95% CI, allowing different sds between groups. For binary variables, the effect size is expressed as odds ratio (OR) with (95% CI). An OR of 1.0 can be interpreted as no difference between patients with and without PVA, an OR less than 1 can be interpreted as better clinical outcomes for patients with PVA, and an OR greater than 1, means that patients with PVA have a worse clinical outcomes. Data reported in a median and an interquartile range are converted to mean and sd using the method by Wan et al (22).

## RESULTS

### Search

**Figure 1.**
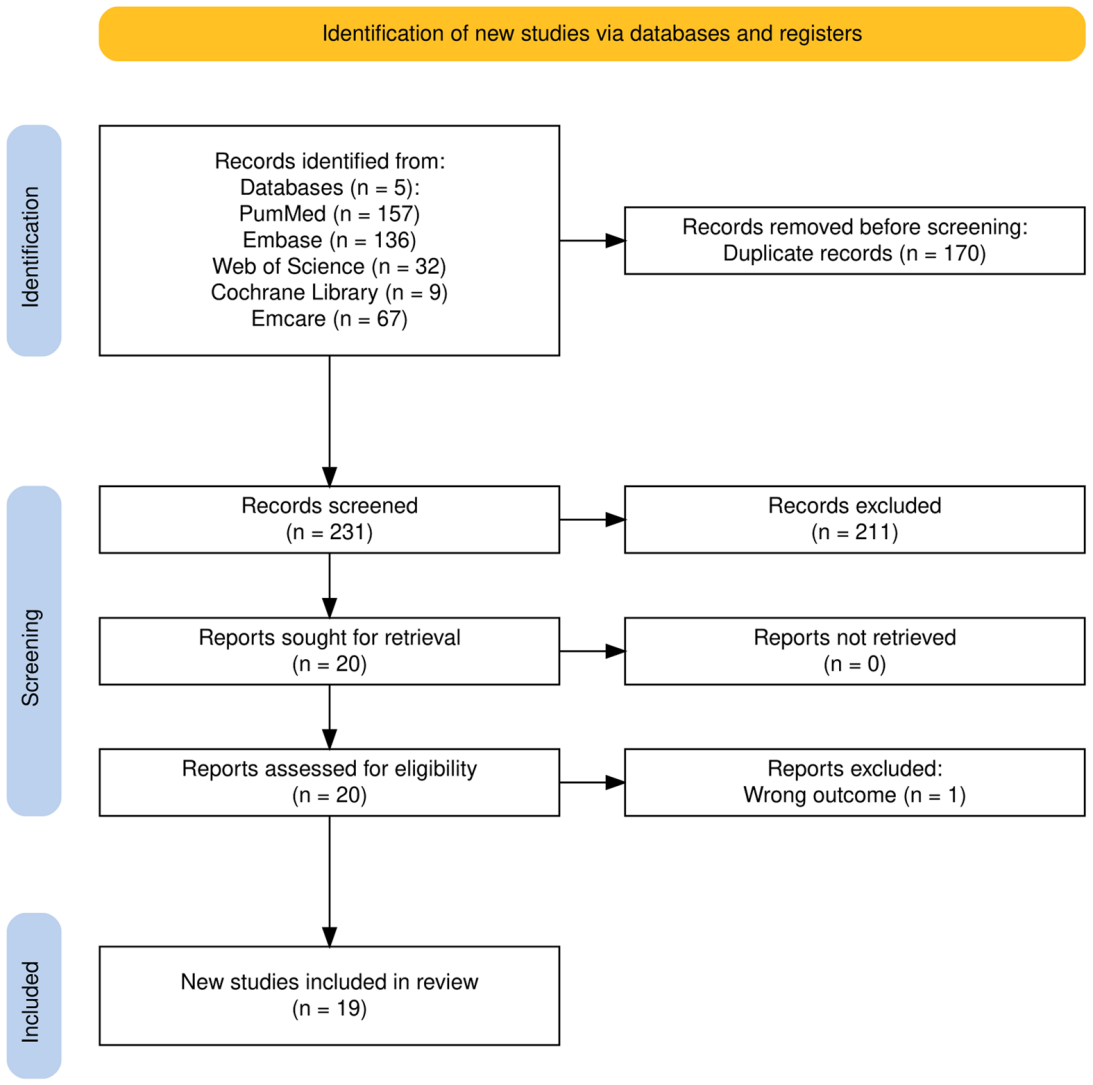
Preferred Reporting Items for Systematic Reviews and Meta-Analyses flow diagram.

After removing duplicates, 231 articles were screened by title and abstract. Twenty full-text articles were read for eligibility. Nineteen articles were suitable based on the inclusion and exclusion criteria and have been included in this systematic review. The flowchart can be seen in **Figure 1**.

### Study Characteristics

Nineteen studies involving a total of 2672 patients were included in this review. **Table 1** presents the characteristics of the included studies. All studies included mechanically ventilated ICU patients. One study, conducted by Chao et al (30), exclusively examined patients with a tracheostoma in a weaning center. Robinson et al (23) and Rodriguez et al (37) focused on mechanically ventilated patients with ARDS and trauma, respectively.

**TABLE 1.** Study Characteristics

Study	Study Design	Asynchrony Types	Clinical Outcomes	Patients (*n*)
Robinson et al (23)	PO	IT, DT, SC, PC	MV, ICU and hospital length of stay, mortality	35
Blanch et al (12)	PO	IT, DT, SC, PC	MV, reintubation, tracheostomy, ICU and hospital mortality	50
Thille et al (16)	PO	IT, DT, AT, SC, PC	MV, tracheostomy, mortality	62
Sousa et al (24)	PO	IT, DT (RT), SC, PC	MV, ventilator-free days, tracheostomy, extubating failure, ICU and hospital length of stay, mortality	125
Rolland-Debord et al (25)	AS	IT, AT, DT, SC, PC	MV, ventilator-free days, ICU and hospital length of stay, mortality	103
Martos-Benítez et al (26)	PO	IT, AT, DT, SC, PC, FS	Ventilator-associated pneumonia, MV, length of ICU stay, ICU mortality	122
See et al (27)	RO	IT, DT, FS	Hospital mortality, sedation-free days, ventilator-free days	280
Gogineni et al (28)	PO	IT, DT, AT, PC, FS, auto positive end-expiratory pressure	Prolonged MV, d	28
Rué et al (29)	PO	IT, DT (RT), SC, PC	Discharge status: dead or alive	139
Chao et al (30)	PO	IT	Weaning success	200
Sadek et al (31)	PO	IT, DT, AT, SC, PC, FS	Weaning success	100
de Wit et al (32)	PO	IT	MV, ICU and hospital mortality, hospital length of stay, reintubation, tracheostomy	60
Magrans et al (33)	PO	IT and DT	Status at ICU discharge; dead or alive	180
Vaporidi et al (34)	PO	IT	MV, length of ICU stay, mortality	110
Ge et al (13)	RO	DT, IT, PC, SC	Ventilator-associated event, mortality	160
Sousa et al (35)	PO	DT (clusters)	MV, ventilator-free days, reintubation, tracheostomy, ICU and hospital length of stay, mortality	103
Zhou et al (2)	RO	DT, IT, SC, PC, FS	Ventilator-free days, MV, lCU and hospital length of stay, mortality	676
Mellado Artigas et al (36)	AS	RT	Extubating rate	39
Rodriguez et al (37)	PO	RT	Discontinuation of MV, 90-d mortality	100

AS = ancillary study, AT = auto-triggering, DT = double triggering, FS = flow starvation, IT = ineffective triggering, MV = mechanical ventilation, PC = prolonged cycling, PO = prospective cohort, RO = retrospective cohort, RT = reverse triggering, SC = short cycling.

IT index = IT events/total number of breaths (including IT).

The results of the Risk of Bias assessment can be seen in **Appendix B** (https://links.lww.com/CCM/H776).

### Different Types of PVA Combined

#### Studies on PVA: Meta-Analysis and Results

Nine studies (12, 16, 23–29) looked at multiple types of asynchrony. Six studies used the AI to indicate the degree of asynchrony defined as number of asynchronous events/total number of breaths (including ineffective triggers) (12, 16, 23–26), three articles (See et al [27], Gogineni et al [28], and Rué et al [29]) that described multiple types of PVA were not included in the meta-analysis due to a different definition of the AI. Specifically, these articles did not use the AI boundary of below and above 10%. As a result, they were not included in the meta-analysis. The characteristics of these studies can be seen in **Table 2**. All six studies included in the meta-analysis looked at ineffective triggering, double triggering, short cycling, and prolonged cycling. Martos-Benítez et al (26) additionally looked at flow-related asynchronies. In Sousa et al (24), the number of double triggering events also included reverse triggering as their method could not make a clear distinction between these asynchronies. A meta-analysis was performed for MV duration (d), length of ICU stay (d), and ICU and hospital mortality. A meta-analysis with the MD was conducted with these six studies. Martos-Benítez et al (26) determined on days 1, 3, 4, and 7, the difference between patients with an AI less than 10% and an AI greater than 10%. Day 3 was considered for this meta-analysis.

**TABLE 2.** Characteristics of Studies That Examined Multiple Types of Patient-Ventilator Asynchrony

Study	Detection Method	Moment of Measurement	Outcome
Robinson et al (23)	Visual inspection by two researchers, with intervention of a third if no agreement	30 min in the first 48 hr of ventilator initiation	MV, ICU and hospital length of stay, mortality
Blanch et al (12)	BetterCare software (36)	Continuously, during the period a patient was on MV^1^	MV, reintubation, tracheostomy, ICU and hospital mortality
Thille et al (16)	Visual inspection by researchers	Once, 30 min after endotracheal suction	MV, tracheostomy, mortality
Sousa et al (24)	BetterCare software (36)	Continuously until liberation from the ventilator, death, tracheostomy, or 28 d of MV	MV, ventilator-free days, tracheostomy, extubating failure, ICU and hospital length of stay, mortality (ICU and hospital)
Rolland-Debord et al (25)	RCR software	20 min. After 12, 24, 36, and 48 hr after inclusion	MV, ventilator-free days, length of ICU and hospital stay, ICU and hospital mortality
Martos-Benítez et al (26)	Visual inspection by two different physicians	30 min every day, for the first 7 d of MV	Ventilator-associated pneumonia, MV, length of ICU stay, ICU mortality

MV = mechanical ventilation.

These studies all divided patients into high degree and low degree of asynchrony, AI index less than 10%, and AI index greater than 10%. These two patient groups were then compared on outcome measures.

**Figure 2.**
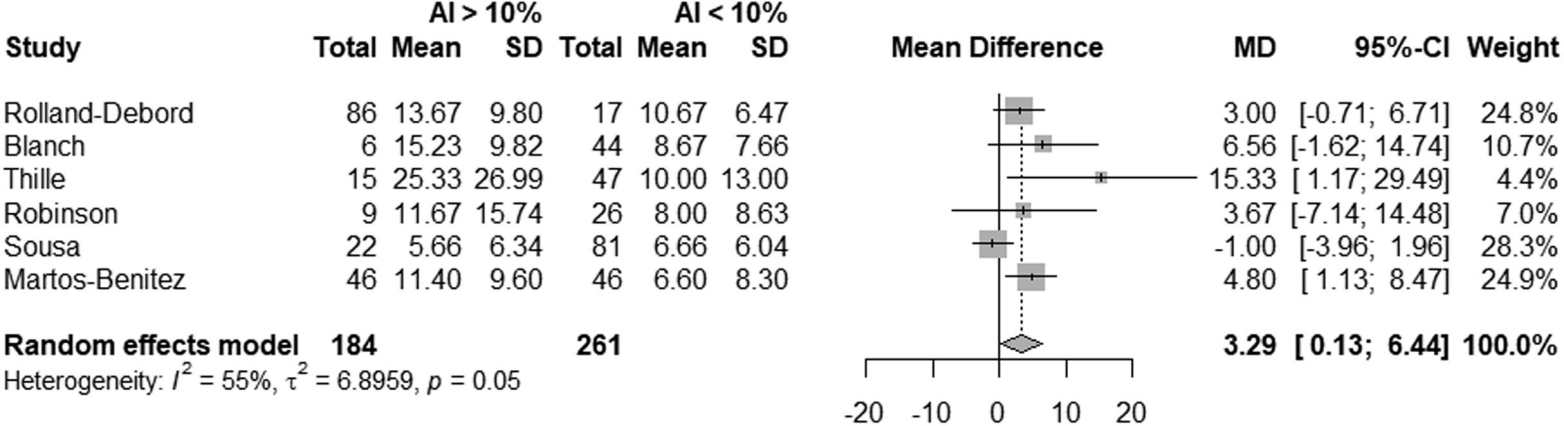
*Forest plot* showing differences in mechanical ventilation duration by asynchrony index (AI) greater than 10%. MD = mean difference (d).

**Figure 3.**
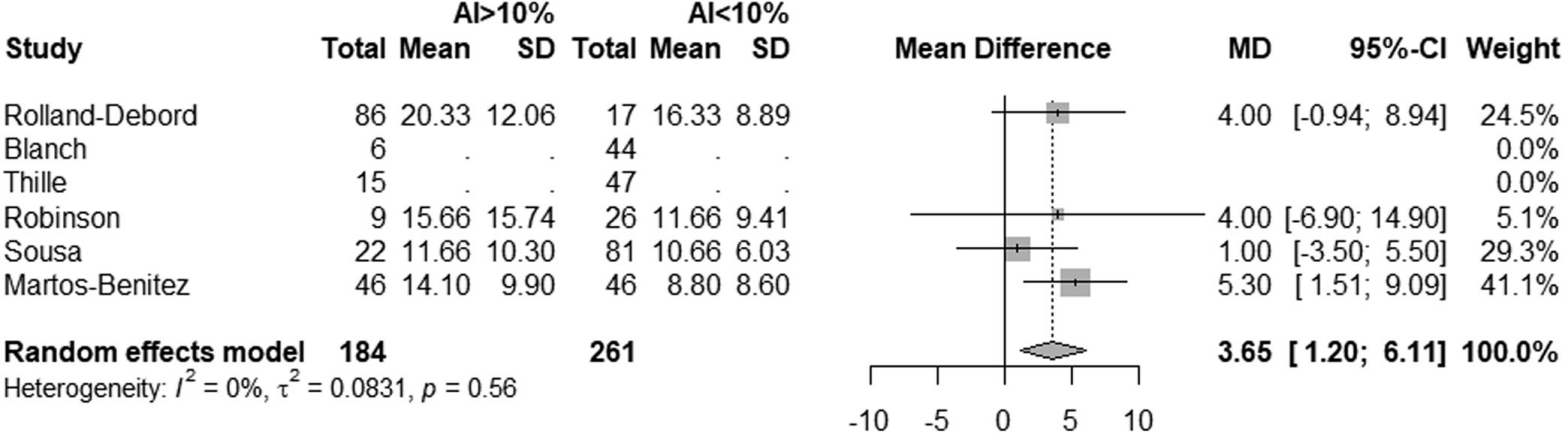
*Forest plot* showing differences in length of stay on the ICU by asynchrony index (AI) greater than 10%. MD = mean difference (d).

The results of the meta-analysis are presented in **Figures 2** and **3** and **Appendices C** and **D** (https://links.lww.com/CCM/H776). These results show a statistically significant difference in MV (d) between patients with an AI index less than 10%, and patients with an AI index greater than 10% (MD, 3.29 d [0.13–6.44 d]) also a statistically significant difference is seen in ICU length of stay (MD, 3.65 d [1.20–6.11 d]). No difference is found in ICU mortality or hospital mortality (OR, 1.63 [0.61–4.35] and OR, 1.50 [0.51–4.35]).

Additionally, another meta-analysis was conducted with four studies that determined the asynchrony of patients in multiple days of ICU admission, including the studies of Blanch et al (12), Sousa et al (24), Rolland-Debord et al (25), and Martos-Benítez et al (26). This resulted in a statistically significantly association between prolonged ICU length of stay and patients with an AI index greater than 10% (MD, 3.61 [0.97–6.26]). No statistically significantly association between prolonged duration of MV and patients with an AI index greater than 10% was seen (MD, 2.63 [–0.60 to 5.85]) (**Appendix E**, https://links.lww.com/CCM/H776).

These results show that an AI greater than 10% is associated with longer MV duration and longer ICU admission, there was no statistically significant association with mortality.

Due to the heterogeneity of the methodology of the remaining three articles, their results will be described in summary form and can be seen in **Table 3**.

**TABLE 3.** Characteristics of Studies Describing Patient-Ventilator Asynchrony

Study	Detection Method	AI	Duration of Measurement	Outcome
See et al (27)	Visual inspection twice a day	Dichotomy: present vs. absent	120 s	Ventilator-free days
Rué et al (29)	Software, study-created algorithm	AI = proportion of asynchronous events among total number of ventilator cycles	Continuously	Mortality
Gogineni et al (28)	Visual inspection daily	Average number of specific pattern of patient-ventilator asynchrony/average number of respiratory efforts over 3 d	60 s	Mechanical ventilation duration

AI = asynchrony index.

The results of the study by See et al (27) show an increased mortality for patients with PVA. No significant difference was found in duration of MV.

Rué et al (29) investigated the impact of PVA on hospital mortality. The findings of the study indicate an association between PVA and a lower hospital mortality.

Gogineni et al (28) looked at the type of asynchrony that best predicts prolonged MV. The results indicate that flow asynchrony best predicts whether patients will have prolonged MV duration.

Below, the clinical outcomes on different subtypes of PVA will be described separately.

#### Ineffective Triggering

Six studies (13, 30–34) described the association between ineffective triggering on the clinical outcome measures. The characteristics of these studies are shown in **Appendix F** (https://links.lww.com/CCM/H776).

The results of the two studies examining the impact of ineffective triggering on weaning success rate indicates that patients with ineffective triggering experience significantly more instances of weaning failure (30, 31).

The results of the study with Ge et al (13) show that there is no association between patients with ineffective triggering and developing a ventilator-associated event (VAE) or mortality.

In addition, two studies examined the effect of the occurrence of ineffective triggering clusters on the clinical outcome measure. Clusters are a certain number of PVAs in a given unit of time. Both studies demonstrated that there is an association between the presence of ineffective triggering clusters and a longer MV duration (33, 34).

The ineffective triggering index (ITI) was described by two studies (32, 34). ITI is defined as the amount of ineffective triggering asynchronies/total number of breaths (including ineffective triggers) × 100%.

Both studies divided patients into two groups, a group of patients with an ITI index less than 10% and a group of patients with an ITI index greater than 10%. The results of de Wit et al (32) demonstrate that there is an association between patients with an ITI greater than 10% and duration of MV (d) and ICU length of stay, but no association between ITI greater than 10% and mortality was found. Results from Vaporidi et al (34) show that no association was found between patients with an ITI greater than 10% and clinical outcome measures; however, they did find an association between patients with clusters of ineffective triggers and clinical outcome measures including duration of MV and hospital mortality.

#### Double Triggering

Three studies investigated an association between the occurrence of double triggering asynchrony and clinical outcome measures (2, 13, 35), their characteristics can be seen in **Appendix G** (https://links.lww.com/CCM/H776). No meta-analysis was performed between these studies due to different measure of double triggering. The results of two studies, Zhou et al (2) and Sousa et al (24) indicate that with double triggering, occurring either in clusters or as single events, there is an association between double triggering and prolonged MV and ICU length of stay (35). Ge et al (13) did not find an association between double triggering and mortality or a VAE.

#### Reverse Triggering

Two studies (36, 37) described the impact of reverse triggering on clinical outcome measures, the characteristics of these studies can be found in **Appendix H** (https://links.lww.com/CCM/H776). The results of Mellado Artigas et al (36) describe the impact of reverse triggering on outcome measures in recently intubated patients with assist-control ventilation in the ICU, a volume-controlled ventilation mode. This shows that patients with reverse triggering switch more quickly to an assisted mode or to extubating in the following 24 hours. Rodriguez et al (37) found a significant reduction in 90-day mortality in patients with ARDS that had reverse triggering. However, no difference was found in extubating success between patients with reverse triggering and patients without reverse triggering.

## DISCUSSION

This systematic review evaluated how PVA relates to clinical outcomes in mechanically ventilated ICU patients. Our findings show there is an association between the presence of different types of asynchronies, specifically ineffective triggering and double triggering, and a prolonged MV duration and extended ICU length of stay. No association was found between PVA and mortality either within the ICU or during hospitalization.

The findings of this study demonstrate that there is an association between patients experiencing more than 10% asynchrony and a prolonged MV and ICU length of stay duration by, respectively, 3 and 3.5 days.

Surprisingly, we found that the presence of reverse triggering without breath stacking had a positive association with clinical outcome measures. The presence of reverse triggering was associated with a higher extubation rate in the following 24 hours (36) and was associated, in another study, with a reduced 90-day mortality (37). Despite this, conflicting results have been reported in the literature regarding the association of reverse triggering on patients’ outcomes. One case study described that reverse triggering without breath stacking leads to an injurious inflation pattern (38). In this study, no effects on clinical outcomes were provided. However, reverse triggering leading to additional insufflation, that is, resulting in double triggering, is known to have an association with adverse effects (39).

In contrast to our findings, Kyo et al (11) reported an association between PVA and increased ICU and hospital mortality. However, their study had low certainty of evidence and did not differentiate between various types of asynchrony, which may explain the differing results regarding mortality. Our review addresses this gap by examining the effects of specific types of asynchrony, providing a more nuanced understanding of their individual impacts on clinical outcomes. Notably, our findings suggest that reverse triggering might have positive effects on clinical outcomes, highlighting the need for detailed analysis of different types of asynchrony.

This review does contain some limitations that should be considered. First, the potential for bias in the included studies should be acknowledged. This bias arises primarily from the lack of adjustment for confounding factors, including variables such as sedation, comorbidities, and the use of different medications. These factors can significantly increase or decrease the occurrence of PVA depending on the type of asynchrony. Some studies included in this review did examine the impact of these factors on outcomes and can be found in **Appendices J** and **K** (https://links.lww.com/CCM/H776). These studies show that different medications, such as midazolam and propofol, may influence the occurrence and severity of PVA, with midazolam associated with increased asynchrony and propofol potentially reducing certain types, such as double triggering (Ge et al [13], Martos-Benítez et al [26]). Additionally, comorbidities such as COPD, ARDS, sepsis, and smoking have been reported to be associated with specific types of asynchrony (Zhou et al [2], Chao et al [30], Vaporidi et al [34]), although findings across studies are variable.

In addition, ventilator mode is an important covariate. The results of the included studies and the results are presented in **Appendix I** (https://links.lww.com/CCM/H776). Future studies should account for these confounding factors. This includes the recommendation that future studies should also investigate whether asynchrony is common in certain groups of patients.

Second, the presence of heterogeneity among the included studies in this review should be taken into account, particularly within the studies included in the meta-analysis that investigated the association between asynchrony and clinical outcome measures. All the studies in the meta-analysis used the AI. However, methodological variation exist in the determination of asynchrony, ranging from continuous software-based measurements to manual analysis of flow, pressure, and volume curves over different time intervals. Additionally, heterogeneity is observed in the types of asynchrony considered among the studies in the meta-analysis. Specifically, the inclusion of different types of asynchronies in the calculation of the AI contributes to this heterogeneity. Furthermore, variations in asynchrony occurrence between day and night contribute to the observed heterogeneity (13). Based on these findings, we recommend that in future studies only continuous monitoring of PVA should be considered.

## CONCLUSIONS

This systematic review highlights the clinical relevance of PVA by demonstrating associations with prolonged MV and ICU length of stay. Despite its limitations, the findings underscore the importance of addressing PVA in clinical practice to improve patient outcomes. This review serves as a hypothesis-generating analysis, suggesting that PVA, particularly ineffective triggering and double triggering, may contribute to worse clinical outcomes. However, due to methodological differences in the studies reviewed, a definitive conclusion regarding double triggering’s association with clinical outcomes cannot be drawn. Further research, with standardized definitions and methodologies, is needed to confirm these associations and explore the mechanisms behind them. Such studies could refine clinical management strategies for PVA.

## ACKNOWLEDGMENTS

We thank Jan Schoones for invaluable help in crafting the search string crucial for this publication.

## Supplementary Material


