# Supplementary material for: The Association Between Patient-Ventilator Asynchrony and Clinical Outcomes in Mechanically Ventilated Patients: A Systematic Review
Source: Crit Care Med. 2025 Aug 12;53(11):e2261–70. doi: 10.1097/CCM.0000000000006816 (PMC12577656; doi:10.1097/CCM.0000000000006816)
Supplement: Supplementary file 1 [file ccm-53-e2261-s001.pdf]

Supplementary information

A. Prisma checklist
B. Results of the QUIPS tool
C. Meta-analysis of PVA on ICU mortality
D. Meta-analysis of PVA on hospital mortality
E. Meta-analysis of studies with continuous monitoring of PVA
F. Characteristics of the studies that describe ineffective triggering
G. Characteristics of studies describing double triggering
H. Characteristics of studies describing reverse triggering
I. Ventilation modes & PVA
J. Medication and PVA
K. Comorbidities & PVA
L. Search string

A. Prisma checklist

Section and Topic	Item #	Checklist item	Location where item is reported
**TITLE**			
Title	1	Identify the report as a systematic review.	Page: 1
**ABSTRACT**			
Abstract	2	See the PRISMA 2020 for Abstracts checklist.	Page: 1,2
**INTRODUCTION**			
Rationale	3	Describe the rationale for the review in the context of existing knowledge.	Page: 2,3
Objectives	4	Provide an explicit statement of the objective(s) or question(s) the review addresses.	Page: 3
**METHODS**			
Eligibility criteria	5	Specify the inclusion and exclusion criteria for the review and how studies were grouped for the syntheses.	Page: 4
Information sources	6	Specify all databases, registers, websites, organisations, reference lists and other sources searched or consulted to identify studies. Specify the date when each source was last searched or consulted.	Page: 5
Search strategy	7	Present the full search strategies for all databases, registers and websites, including any filters and limits used.	Supplementary: pages: 8-12
Selection process	8	Specify the methods used to decide whether a study met the inclusion criteria of the review, including how many reviewers screened each record and each report retrieved, whether they worked independently, and if applicable, details of automation tools used in the process.	Page: 5
Data collection process	9	Specify the methods used to collect data from reports, including how many reviewers collected data from each report, whether they worked independently, any processes for obtaining or confirming data from study investigators, and if applicable, details of automation tools used in the process.	Page: 5
Data items	10a	List and define all outcomes for which data were sought. Specify whether all results that were compatible with each outcome domain in each study were sought (e.g. for all measures, time points, analyses), and if not, the methods used to decide which results to collect.	Page: 4/5
	10b	List and define all other variables for which data were sought (e.g. participant and intervention characteristics, funding sources). Describe any assumptions made about any missing or unclear information.	Page: 4/5
Study risk of bias assessment	11	Specify the methods used to assess risk of bias in the included studies, including details of the tool(s) used, how many reviewers assessed each study and whether they worked independently, and if applicable, details of automation tools used in the process.	Page: 5
Effect measures	12	Specify for each outcome the effect measure(s) (e.g. risk ratio, mean difference) used in the synthesis or presentation of results.	Page 5/6
Synthesis methods	13a	Describe the processes used to decide which studies were eligible for each synthesis (e.g. tabulating the study intervention characteristics and comparing against the planned groups for each synthesis (item #5)).	
	13b	Describe any methods required to prepare the data for presentation or synthesis, such as handling of missing summary statistics, or data conversions.	
	13c	Describe any methods used to tabulate or visually display results of individual studies and syntheses.	
	13d	Describe any methods used to synthesize results and provide a rationale for the choice(s). If meta-analysis was performed, describe the model(s), method(s) to identify the presence and extent of statistical heterogeneity, and software package(s) used.	Page 5/6
	13e	Describe any methods used to explore possible causes of heterogeneity among study results (e.g. subgroup analysis, meta-regression).	Page 5/6
	13f	Describe any sensitivity analyses conducted to assess robustness of the synthesized results.	
Reporting bias assessment	14	Describe any methods used to assess risk of bias due to missing results in a synthesis (arising from reporting biases).	

Section and Topic	Item #	Checklist item	Location where item is reported
Certainty assessment	15	Describe any methods used to assess certainty (or confidence) in the body of evidence for an outcome.	
**RESULTS**			
Study selection	16a	Describe the results of the search and selection process, from the number of records identified in the search to the number of studies included in the review, ideally using a flow diagram.	Figure 1
	16b	Cite studies that might appear to meet the inclusion criteria, but which were excluded, and explain why they were excluded.	Figure 1
Study characteristics	17	Cite each included study and present its characteristics.	Table
Risk of bias in studies	18	Present assessments of risk of bias for each included study.	Supplementary
Results of individual studies	19	For all outcomes, present, for each study: (a) summary statistics for each group (where appropriate) and (b) an effect estimate and its precision (e.g. confidence/credible interval), ideally using structured tables or plots.	Table
Results of syntheses	20a	For each synthesis, briefly summarise the characteristics and risk of bias among contributing studies.	Table
	20b	Present results of all statistical syntheses conducted. If meta-analysis was done, present for each the summary estimate and its precision (e.g. confidence/credible interval) and measures of statistical heterogeneity. If comparing groups, describe the direction of the effect.	
	20c	Present results of all investigations of possible causes of heterogeneity among study results.	
	20d	Present results of all sensitivity analyses conducted to assess the robustness of the synthesized results.	
Reporting biases	21	Present assessments of risk of bias due to missing results (arising from reporting biases) for each synthesis assessed.	
Certainty of evidence	22	Present assessments of certainty (or confidence) in the body of evidence for each outcome assessed.	
**DISCUSSION**			
Discussion	23a	Provide a general interpretation of the results in the context of other evidence.	Pages: 10-12
	23b	Discuss any limitations of the evidence included in the review.	Pages: 11-12
	23c	Discuss any limitations of the review processes used.	Pages: 11-12
	23d	Discuss implications of the results for practice, policy, and future research.	Pages: 10-12
**OTHER INFORMATION**			
Registration and protocol	24a	Provide registration information for the review, including register name and registration number, or state that the review was not registered.	
	24b	Indicate where the review protocol can be accessed, or state that a protocol was not prepared.	
	24c	Describe and explain any amendments to information provided at registration or in the protocol.	
Support	25	Describe sources of financial or non-financial support for the review, and the role of the funders or sponsors in the review.	
Competing interests	26	Declare any competing interests of review authors.	
Availability of data, code and other materials	27	Report which of the following are publicly available and where they can be found: template data collection forms; data extracted from included studies; data used for all analyses; analytic code; any other materials used in the review.	

From: Page MJ, McKenzie JE, Bossuyt PM, Boutron I, Hoffmann TC, Mulrow CD, et al. The PRISMA 2020 statement: an updated guideline for reporting systematic reviews. BMJ 2021;372:n71. doi: 10.1136/bmj.n71

B. Results of the QUIPS tool

Table 1. *Results of the QUIPS tool*

Study	Study Participation	Study Attrition	Prognostic Factor Measurement	Outcome Measurement	Study Confounding	Statistical Analysis and Reporting
Artigas (2021)	Moderate	Moderate	Low	Moderate	High	Low
Blanch (2015)	Low	Low	Low	Moderate	High	Moderate
Chao (1997)	Low	Moderate	Low	Moderate	High	Low
de Wit (2009)	Low	Low	Low	Low	Low	Low
Ge (2020)	Low	Moderate	Low	Low	Low	Low
Gogineni (2012)	Low	Moderate	Moderate	Low	Low	Moderate
Magrans (2022)	Moderate	Moderate	Low	Low	Moderate	Low
Martos-Benitez (2020)	Low	High	Low	Low	Low	Low
Robinson (2013)	Low	Low	Low	Low	Moderate	Moderate
Rodriguez (2021)	Low	Moderate	Low	Low	Low	Low
Rolland-Debord (2017)	Low	Moderate	Low	Moderate	Low	Low
Rue (2017)	High	Moderate	Moderate	Low	High	Moderate
Sadek (2021)	Low	High	Low	Low	Moderate	Low
See (2020)	Low	High	Moderate	Low	Low	Moderate
Sousa (2020)	Low	Moderate	Low	Low	Low	Low
Sousa (2021)	Low	Moderate	Low	Low	Low	Low
Thille (2006)	Low	Moderate	Low	Moderate	Moderate	Low
Vaporidi (2017)	Low	High	Low	Low	Low	Moderate
Zhou (2021)	Low	High	Low	Low	Low	Low

C. Meta-analysis of PVA on ICU mortality

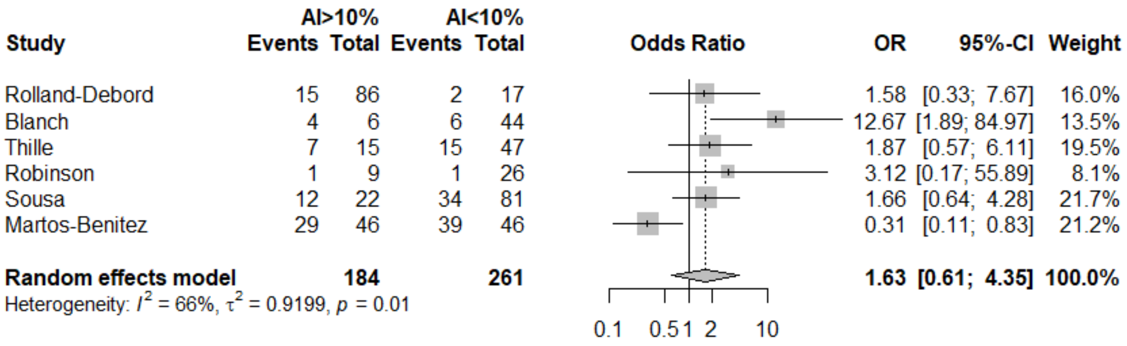

Figure 3. *Forest plot of intensive care unit (ICU) mortality, AI >10%. AI = Asynchrony Index, OR = odds ratio*

D. Meta-analysis of PVA on hospital mortality

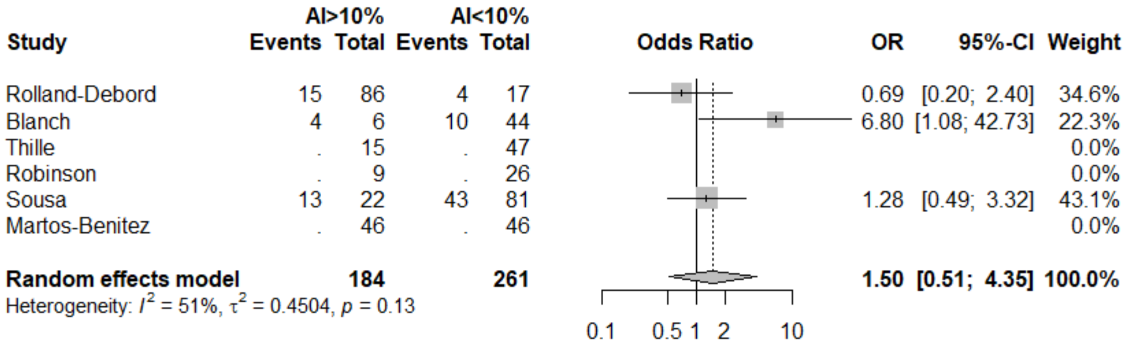

Figure 4. *Forest plot of hospital mortality, AI >10%. AI = Asynchrony index, OR = odds ratio*

E. Meta-analysis of studies with continuous monitoring of PVA

E1. ICU length of stay

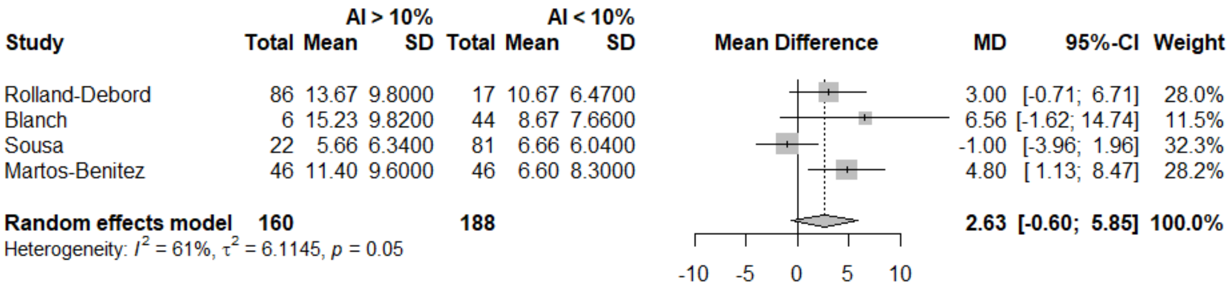

Figure 2. *Meta-analysis of the effect of PVA on the ICU length of stay. MV = Mechanical Ventilation*

E2. Mechanical ventilation duration

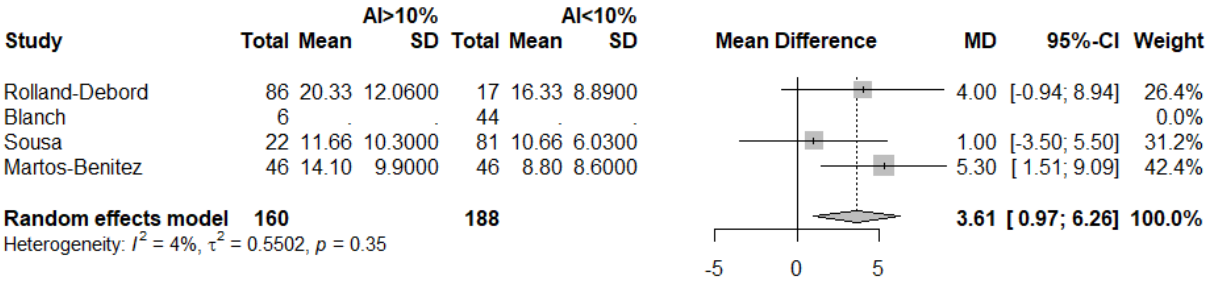

Figure 3. *Meta-analysis of MV-duration. MV = mechanical ventilation*

F. Characteristics of the studies that describe ineffective triggering

Table 2. *Characteristics of the studies describing ineffective triggering*

Study	Detection Method	Asynchrony index	Duration of measurement	Outcome
Chao (22)	Visual inspection of accessory respiratory muscles, and visual inspection of the monitor	Prevalence of IT	Minimum observation time of two minutes	Weaning success
Sadek (31)	Visual inspection	Asynchronous events/total number of breaths (including IT).	20 minutes after 12-, 24-, 36-, and 48-hours following intubation	Weaning success
De Wit (32)	Visual inspection by two researchers, if no agreement could be reached, a third reviewer was requested.	Ineffective trigger index (ITI) = total amounts of IT / total breaths (including IT)	10-minute observation within the first 24 hours of MV.	MV, ICU- and hospital mortality, hospital length of stay, reintubation, tracheostomy
Magrans (33)	BetterCare Software (37)	> 10% breaths with IT (cluster)	Continuously, throughout the MV period.	Status at ICU discharge dead or alive
Vaporidi (34)	Software, with a prototype monitor (40)	>30 IT in a 3-min period (cluster) & ITI	Continuously with software for 24 hours on day 1, 3 and 6.	MV, length of ICU stay, mortality
Ge (10)	Software. Study-created deep learning algorithm	Asynchronous events/total number of breaths (including IT)	Continuously, throughout the MV period.	Ventilator associated event, mortality

IT = Ineffective triggering, DT = double triggering, ITI = ineffective trigger index, MV = mechanical ventilation

G. Characteristics of studies describing double triggering

Table 3. *Characteristics of studies describing double triggering*

Study	Detection Method	Duration of measurement	Asynchrony index	Outcomes
Sousa (2021) (35)	BetterCare software (37)	Continuously throughout MV-period	Asynchronous events/total number of breaths (including IT)	MV, ventilator-free days, reintubation, tracheostomy, ICU and hospital length of stay, mortality.
Zhou (2)	Visual inspection	10-minute assessment every four hours	> 6 double triggering events in 3-min period (cluster)	Ventilator-free days, MV, ICU and hospital length of stay, ICU, and hospital mortality
Ge (10)	Software. Study-created deep learning algorithm	Continuously throughout MV-period	Prevalence of every type of PVA	Ventilator associated event, mortality

MV = mechanical ventilation, PVA = patient-ventilator asynchrony, IT = ineffective triggering

H. Characteristics of studies describing reverse triggering

Table 4. *Characteristics of studies describing reverse triggering*

Study	Detection Method	Duration of measurement	Asynchrony index	Outcomes
Artigas (36)	Visually reviewed by three reviewers and with the use of a detection algorithm	One hour recording 24 hours after intubation	> 10% of total breaths with RT	Extubating rate
Rodriguez (24)	Detection software	30 min	Total RT / minute	Discontinuation of MV1, 90-days mortality

RT = reverse triggering, MV = mechanical ventilation

I. Ventilation modes & PVA

Table 5. *Effects of ventilation mode on PVA*

Study	Ventilation mode	Outcome
Ge et al.	PCV vs PSV	DT more In PCV than in PSV, IT more in PSV than in PCV
Zhou et al.	PC vs VC	VC more DT and flow mismatch
Sousa et al.	Assisted vs control	PVA more in assisted mode, DT more in controlled mode
Blanch et al.	PSV vs VCV vs PCV	DT more in PCV and PSV vs VCV, IT more in PSV than in VCV or PCV. Median AI not significant different.
Robinson et al.	SIMV + PSV vs SIMV	AI > 10%, most use of SIMV + PSV

PCV = pressure control ventilation, PSV = pressure support ventilation, PC= pressure control, VC = volume control, NAVA = Neurally Adjusted Ventilatory Assist, VCV = volume control ventilation, SIMV = synchronized intermittent mandatory ventilation, DT = double triggering, IT = ineffective triggering, AI = asynchrony index, PVA = patient ventilator asynchrony

J. Medication and PVA

Table 6. *Effects of medication on PVA*

Study	Medication	Outcome
Ge et al.	Propofol	Propofol reduced incidence of DT 30-60 min after exposure, increased after 2-4 hours.
Rodriguez	Fentanyl	Patients with RT, had a lower fentanyl infusion rate
Artigas	Propofol, midazolam, fentanyl, hydromorphone	No difference between the groups
Martos-Benitez	Midazolam, propofol, ketamine	Frequency of IT significantly higher in patients with deep sedation, starved flow and short cycling associated with agitated patients. Midazolam associated with higher frequency of severe PVA, propofol associated with a lower frequency of PVA. Use and dose of fentanyl, ketamine not significantly related to severe PVA

DT = double triggering, RT = reverse triggering, IT = ineffective triggering, PVA = patient ventilator asynchrony

K. Comorbidities & PVA

Table 7. *Effects of comorbidities on PVA*

Study	Comorbidity	Outcome
Rodriguez	ARDS, APACHE II	RT associated with greater ARDS severity (p=0.08), lower APACHE II score (p=0.052)
Vaporidi	Sepsis, APACHE II	Patients with sepsis had more events. No difference seen in APACHE II score.
De Wit	COPD, APACHE II	No differences between groups
Chao	COPD	Patients with IT had significantly more COPD
Robinson	COPD	No differences in COPD between both groups
Sadek	Smoking	Smoking and APACHE II score higher among those with failed weaning
Gogineni	APACHE II	No differences found between both groups
Thille	SOFA, SAPS II, COPD	IT more COPD, SAPS II, and SOFA no difference
Zhou	Smoking, cirrhosis, pneumonia, sepsis, ARDS, heart disease,	Smoking, cirrhosis, pneumonia, sepsis, and ARDS risk factor for PVA, heart disease negatively associated with overall PVA
Sousa	ARDS, smoking, SAPS 3	Higher SAPS 3 associated with higher AI

ARDS = Acute respiratory distress syndrome, APACHE II = Acute Physiology and Chronic Health Evaluation II, COPD = chronic obstructive pulmonary disease, SOFA = Sequential Organ Failure Assessment, SAPS = Simplified Acute Physiology Score

L. Search string

PubMed

http://www.ncbi.nlm.nih.gov/pubmed?otool=leiden

1-2-2023

(("patient ventilator asynchrony"[ti] OR "patient ventilator asynchronies"[ti] OR "patient ventilator asynchron*"[ti] OR "patient ventilation asynchrony"[ti] OR "ventilator asynchrony"[ti] OR "ventilator asynchronies"[ti] OR "ventilator asynchron*"[ti] OR "asynchronous ventilation"[ti] OR "asynchronous ventilations"[ti] OR "ventilator asynchrony"[title:~6] OR "ventilator asynchronies"[title:~6] OR "ventilator asynchronic"[title:~6] OR "ventilators asynchrony"[title:~6] OR "ventilators asynchronies"[title:~6] OR "ventilators asynchronic"[title:~6] OR "ventilated asynchrony"[title:~6] OR "ventilated asynchronies"[title:~6] OR "ventilated asynchronic"[title:~6] OR "ventilation asynchrony"[title:~6] OR "ventilation asynchronies"[title:~6] OR "ventilation asynchronic"[title:~6] OR "asynchronous ventilation"[title:~6] OR "asynchronous ventilations"[title:~6] OR "asynchron*"[ti] **OR "ineffective efforts"[ti] OR "ineffective effort"[ti] OR "ineffective trigger"[ti] OR "ineffective triggers"[ti] OR "ineffective triggering"[ti] OR "ineffective trigger*"[ti] OR "Double triggering"[ti] OR "Reverse triggering"[ti] OR "Auto-triggering"[ti] OR "Premature cycling"[ti] OR "Delayed cycling"[ti] OR "Flow asynchrony"[ti] OR "flow starvation"[ti] OR "Double triggering"[title:~6] OR "Reverse triggering"[title:~6] OR "Auto-triggering"[title:~6] OR "Premature cycling"[title:~6] OR "Delayed cycling"[title:~6] OR "Flow asynchrony"[title:~6] OR "flow starvation"[title:~6]**) AND ("Respiration, Artificial"[majr] OR "mechanical ventilation"[ti] OR "mechanical ventilations"[ti] OR "Artificial Respiration"[ti] OR "Artificial Respirations"[ti] OR "High-Frequency Ventilation"[ti] OR "High-Frequency Jet Ventilation"[ti] OR "Interactive Ventilatory Support"[ti] OR "Liquid Ventilation"[ti] OR "Noninvasive Ventilation"[ti] OR "One-Lung Ventilation"[ti] OR "Positive-Pressure Respiration"[ti] OR "Continuous Positive Airway Pressure"[ti] OR "Intermittent Positive-Pressure Breathing"[ti] OR "Intermittent Positive-Pressure Ventilation"[ti] OR "Ventilator Weaning"[ti] OR "Ventilators, Mechanical"[majr] OR "mechanical ventilator"[ti] OR "mechanical ventilator"[ti] OR "mechanically ventilated"[ti] OR "artificially respirated"[ti] OR "artificially ventilated"[ti] OR "ventilat*"[ti] OR "**pressure-support"[ti] OR** "**pressure-supported"[ti] OR** "**pressure-support*"[ti]**) NOT ("Animals"[mesh] NOT "Humans"[mesh]) AND ("Treatment Outcome"[Mesh] OR "Treatment Outcome"[tw] OR "Outcome"[tw] OR "Outcomes"[tw] OR "Clinical Effectiveness"[tw] OR "Clinical Efficacy"[tw] OR "Treatment Effectiveness"[tw] OR "Treatment Efficacy"[tw] OR "Treatment Failure"[tw] OR "Outcome Assessment, Health Care"[Mesh] OR "Intensive Care Units"[mesh] OR "Intensive Care"[all fields] OR "ICU"[all fields] OR "Critical Care"[mesh] OR "Critical Care"[all fields] OR "Critical Illness"[mesh] OR "critical illness"[all fields] OR "critically ill"[all fields]) **NOT ("Noninvasive Ventilation"[majr] OR "Liquid Ventilation"[majr] OR "High-Frequency Jet Ventilation"[majr] OR "One-Lung Ventilation"[majr] OR "Non-invasive Ventilation"[ti] OR "Noninvasive Ventilation"[ti] OR "Liquid Ventilation"[ti] OR "High-Frequency Jet Ventilation"[ti] OR "One-Lung Ventilation"[ti] OR "Non-invasive Ventilat*"[ti] OR "Noninvasive Ventilat*"[ti] OR "Liquid Ventilat*"[ti] OR "High-Frequency Jet Ventilat*"[ti] OR "One-Lung Ventilat*"[ti] OR "Non-invasive Respirat*"[ti] OR "Noninvasive Respirat*"[ti] OR "High-Frequency Jet Respirat*"[ti] OR "One-Lung Respirat*"[ti] OR "Noninvasive"[ti])**)

Embase

http://ovidsp.ovid.com/ovidweb.cgi?T=JS&PAGE=main&MODE=ovid&D=oemezd

(("patient ventilator asynchrony".ti OR "patient ventilator asynchronies".ti OR "patient ventilator asynchron*".ti OR "patient ventilation asynchrony".ti OR "ventilator asynchrony".ti OR "ventilator asynchronies".ti OR "ventilator asynchron*".ti OR "asynchronous ventilation".ti OR "asynchronous ventilations".ti OR ("ventilator" ADJ6 "asynchrony").ti OR ("ventilator" ADJ6 "asynchronies").ti OR ("ventilator" ADJ6 "asynchronic").ti OR ("ventilators" ADJ6 "asynchrony").ti OR ("ventilators" ADJ6 "asynchronies").ti OR ("ventilators" ADJ6 "asynchronic").ti OR ("ventilated" ADJ6 "asynchrony").ti OR ("ventilated" ADJ6 "asynchronies").ti OR ("ventilated" ADJ6 "asynchronic").ti OR ("ventilation" ADJ6 "asynchrony").ti OR ("ventilation" ADJ6 "asynchronies").ti OR ("ventilation" ADJ6 "asynchronic").ti OR ("asynchronous" ADJ6 "ventilation").ti OR ("asynchronous" ADJ6 "ventilations").ti **OR "ineffective efforts".ti OR "ineffective effort".ti OR "ineffective trigger".ti OR "ineffective triggers".ti OR "ineffective triggering".ti OR "ineffective trigger*".ti OR "Double triggering".ti OR "Reverse triggering".ti OR "Auto-triggering".ti OR "Premature cycling".ti OR "Delayed cycling".ti OR "Flow asynchrony".ti OR "flow starvation".ti OR (("Double" ADJ6 "triggering") OR ("Reverse" ADJ6 "triggering") OR ("Premature" ADJ6 "cycling") OR ("Delayed" ADJ6 "cycling") OR ("Flow" ADJ6 "asynchrony") OR ("flow" ADJ6 "starvation")).ti**) AND (exp *"Artificial Respiration"/ OR "mechanical ventilation".ti OR "mechanical ventilations".ti OR "Artificial Respiration".ti OR "Artificial Respirations".ti OR "High-Frequency Ventilation".ti OR "High-Frequency Jet Ventilation".ti OR "Interactive Ventilatory Support".ti OR "Liquid Ventilation".ti OR "Noninvasive Ventilation".ti OR "One-Lung Ventilation".ti OR "Positive-Pressure Respiration".ti OR "Continuous Positive Airway Pressure".ti OR "Intermittent Positive-Pressure Breathing".ti OR "Intermittent Positive-Pressure Ventilation".ti OR "Ventilator Weaning".ti OR *"mechanical ventilator"/ OR "mechanical ventilator".ti OR "mechanical ventilator".ti OR "mechanically ventilated".ti OR "artificially respirated".ti OR "artificially ventilated".ti **OR "pressure-support".ti OR "pressure-supported".ti OR "pressure-support*".ti**) NOT (exp "Animals"/ NOT exp "Humans"/) AND (exp "Treatment Outcome"/ OR "Treatment Outcome".mp OR "Outcome".mp OR "Outcomes".mp OR "Clinical Effectiveness".mp OR "Clinical Efficacy".mp OR "Treatment Effectiveness".mp OR "Treatment Efficacy".mp OR "Treatment Failure".mp OR exp "Intensive Care Unit"/ OR "Intensive Care".af OR "ICU".af OR exp "Intensive Care"/ OR "Critical Care".af OR "Critical Illness"/ OR "critical illness".af OR "critically ill".af) **NOT (exp *"Noninvasive Ventilation"/ OR exp *"Liquid Ventilation"/ OR exp *"High Frequency Jet Ventilation"/ OR exp *"One-Lung Ventilation"/ OR "Non-invasive Ventilation".ti OR "Noninvasive Ventilation".ti OR "Liquid Ventilation".ti OR "High-Frequency Jet Ventilation".ti OR "One-Lung Ventilation".ti OR "Non-invasive Ventilat*".ti OR "Noninvasive Ventilat*".ti OR "Liquid Ventilat*".ti OR "High-Frequency Jet Ventilat*".ti OR "One-Lung Ventilat*".ti OR "Non-invasive Respirat*".ti OR "Noninvasive Respirat*".ti OR "High-Frequency Jet Respirat*".ti OR "One-Lung Respirat*".ti OR Noninvasive.ti)**) NOT (conference review or conference abstract).pt

Web of Science

http://isiknowledge.com/wos

(TI=("patient ventilator asynchrony" OR "patient ventilator asynchronies" OR "patient ventilator asynchron*" OR "patient ventilation asynchrony" OR "ventilator asynchrony" OR "ventilator asynchronies" OR "ventilator asynchron*" OR "asynchronous ventilation" OR "asynchronous ventilations" OR ("ventilator" NEAR/6 "asynchrony") OR ("ventilator" NEAR/6 "asynchronies") OR ("ventilator" NEAR/6 "asynchronic") OR ("ventilators" NEAR/6 "asynchrony") OR ("ventilators" NEAR/6 "asynchronies") OR ("ventilators" NEAR/6 "asynchronic") OR ("ventilated" NEAR/6 "asynchrony") OR ("ventilated" NEAR/6 "asynchronies") OR ("ventilated" NEAR/6 "asynchronic") OR ("ventilation" NEAR/6 "asynchrony") OR ("ventilation" NEAR/6 "asynchronies") OR ("ventilation" NEAR/6 "asynchronic") OR ("asynchronous" NEAR/6 "ventilation") OR ("asynchronous" NEAR/6 "ventilations") **OR "ineffective efforts" OR "ineffective effort" OR "ineffective trigger" OR "ineffective triggers" OR "ineffective triggering" OR "ineffective trigger*" OR "Double triggering" OR "Reverse triggering" OR "Auto-triggering" OR "Premature cycling" OR "Delayed cycling" OR "Flow asynchrony" OR "flow starvation" OR (("Double" NEAR/6 "triggering") OR ("Reverse" NEAR/6 "triggering") OR ("Premature" NEAR/6 "cycling") OR ("Delayed" NEAR/6 "cycling") OR ("Flow" NEAR/6 "asynchrony") OR ("flow" NEAR/6 "starvation")))**) AND TI=("Artificial Respiration" OR "mechanical ventilation" OR "mechanical ventilations" OR "Artificial Respiration" OR "Artificial Respirations" OR "High-Frequency Ventilation" OR "High-Frequency Jet Ventilation" OR "Interactive Ventilatory Support" OR "Liquid Ventilation" OR "Noninvasive Ventilation" OR "One-Lung Ventilation" OR "Positive-Pressure Respiration" OR "Continuous Positive Airway Pressure" OR "Intermittent Positive-Pressure Breathing" OR "Intermittent Positive-Pressure Ventilation" OR "Ventilator Weaning" OR "mechanical ventilator" OR "mechanical ventilator" OR "mechanical ventilator" OR "mechanically ventilated" OR "artificially respirated" OR "artificially ventilated" **OR "pressure-support" OR "pressure-supported" OR "pressure-support*"**) NOT TI=("veterinary" OR "rabbit" OR "rabbits" OR "animal" OR "animals" OR "mouse" OR "mice" OR "rodent" OR "rodents" OR "rat" OR "rats" OR "pig" OR "pigs" OR "porcine" OR "horse" OR "horses" OR "equine" OR "cow" OR "cows" OR "bovine" OR "goat" OR "goats" OR "sheep" OR "ovine" OR "canine" OR "dog" OR "dogs" OR "feline" OR "cat" OR "cats") AND TS=("Treatment Outcome" OR "Treatment Outcome" OR "Outcome" OR "Outcomes" OR "Clinical Effectiveness" OR "Clinical Efficacy" OR "Treatment Effectiveness" OR "Treatment Efficacy" OR "Treatment Failure" OR "Intensive Care Unit" OR "Intensive Care" OR "ICU" OR "Intensive Care" OR "Critical Care" OR "Critical Illness" OR "critical illness" OR "critically ill") **NOT TI=("Non-invasive Ventilation" OR "Noninvasive Ventilation" OR "Liquid Ventilation" OR "High-Frequency Jet Ventilation" OR "One-Lung Ventilation" OR "Non-invasive Ventilat*" OR "Noninvasive Ventilat*" OR "Liquid Ventilat*" OR "High-Frequency Jet Ventilat*" OR "One-Lung Ventilat*" OR "Non-invasive Respirat*" OR "Noninvasive Respirat*" OR "High-Frequency Jet Respirat*" OR "One-Lung Respirat*" OR "Noninvasive")**) NOT DT=(meeting abstract)

Cochrane

https://www.cochranelibrary.com/advanced-search/search-manager

(("patient ventilator asynchrony" OR "patient ventilator asynchronies" OR "patient ventilator asynchron*" OR "patient ventilation asynchrony" OR "ventilator asynchrony" OR "ventilator asynchronies" OR "ventilator asynchron*" OR "asynchronous ventilation" OR "asynchronous ventilations" OR ("ventilator" NEAR/6 "asynchrony") OR ("ventilator" NEAR/6 "asynchronies") OR ("ventilator" NEAR/6 "asynchronic") OR ("ventilators" NEAR/6 "asynchrony") OR ("ventilators" NEAR/6 "asynchronies") OR ("ventilators" NEAR/6 "asynchronic") OR ("ventilated" NEAR/6 "asynchrony") OR ("ventilated" NEAR/6 "asynchronies") OR ("ventilated" NEAR/6 "asynchronic") OR ("ventilation" NEAR/6 "asynchrony") OR ("ventilation" NEAR/6 "asynchronies") OR ("ventilation" NEAR/6 "asynchronic") OR ("asynchronous" NEAR/6 "ventilation") OR ("asynchronous" NEAR/6 "ventilations") **OR "ineffective efforts" OR "ineffective effort" OR "ineffective trigger" OR "ineffective triggers" OR "ineffective triggering" OR "ineffective trigger*" OR "Double triggering" OR "Reverse triggering" OR "Auto-triggering" OR "Premature cycling" OR "Delayed cycling" OR "Flow asynchrony" OR "flow starvation" OR (("Double" NEAR/6 "triggering") OR ("Reverse" NEAR/6 "triggering") OR ("Premature" NEAR/6 "cycling") OR ("Delayed" NEAR/6 "cycling") OR ("Flow" NEAR/6 "asynchrony") OR ("flow" NEAR/6 "starvation")))**):ti AND ("Artificial Respiration" OR "mechanical ventilation" OR "mechanical ventilations" OR "Artificial Respiration" OR "Artificial Respirations" OR "High Frequency Ventilation" OR "High Frequency Jet Ventilation" OR "Interactive Ventilatory Support" OR "Liquid Ventilation" OR "Noninvasive Ventilation" OR "One Lung Ventilation" OR "Positive Pressure Respiration" OR "Continuous Positive Airway Pressure" OR "Intermittent Positive Pressure Breathing" OR "Intermittent Positive Pressure Ventilation" OR "Ventilator Weaning" OR "mechanical ventilator" OR "mechanical ventilator" OR "mechanical ventilator" OR "mechanically ventilated" OR "artificially respirated" OR "artificially ventilated" **OR "pressure-support" OR "pressure-supported" OR "pressure-support*"**):ti)

NOT

("Non-invasive Ventilation" OR "Noninvasive Ventilation" OR "Liquid Ventilation" OR "High-Frequency Jet Ventilation" OR "One-Lung Ventilation" OR "Non-invasive Ventilat*" OR "Noninvasive Ventilat*" OR "Liquid Ventilat*" OR "High-Frequency Jet Ventilat*" OR "One-Lung Ventilat*" OR "Non-invasive Respirat*" OR "Noninvasive Respirat*" OR "High-Frequency Jet Respirat*" OR "One-Lung Respirat*" OR "Noninvasive"):ti

Emcare
http://ovidsp.ovid.com/ovidweb.cgi?T=JS&NEWS=n&CSC=Y&PAGE=main&D=emcr

(("patient ventilator asynchrony".ti OR "patient ventilator asynchronies".ti OR "patient ventilator asynchron*".ti OR "patient ventilation asynchrony".ti OR "ventilator asynchrony".ti OR "ventilator asynchronies".ti OR "ventilator asynchron*".ti OR "asynchronous ventilation".ti OR "asynchronous ventilations".ti OR ("ventilator" ADJ6 "asynchrony").ti OR ("ventilator" ADJ6 "asynchronies").ti OR ("ventilator" ADJ6 "asynchronic").ti OR ("ventilators" ADJ6 "asynchrony").ti OR ("ventilators" ADJ6 "asynchronies").ti OR ("ventilators" ADJ6 "asynchronic").ti OR ("ventilated" ADJ6 "asynchrony").ti OR ("ventilated" ADJ6 "asynchronies").ti OR ("ventilated" ADJ6 "asynchronic").ti OR ("ventilation" ADJ6 "asynchrony").ti OR ("ventilation" ADJ6 "asynchronies").ti OR ("ventilation" ADJ6 "asynchronic").ti OR ("asynchronous" ADJ6 "ventilation").ti OR ("asynchronous" ADJ6 "ventilations").ti **OR "ineffective efforts".ti OR "ineffective effort".ti OR "ineffective trigger".ti OR "ineffective triggers".ti OR "ineffective triggering".ti OR "ineffective trigger*".ti** **OR "Double triggering".ti OR "Reverse triggering".ti OR "Auto-triggering".ti OR "Premature cycling".ti OR "Delayed cycling".ti OR "Flow asynchrony".ti OR "flow starvation".ti OR (("Double" ADJ6 "triggering") OR ("Reverse" ADJ6 "triggering") OR ("Premature" ADJ6 "cycling") OR ("Delayed" ADJ6 "cycling") OR ("Flow" ADJ6 "asynchrony") OR ("flow" ADJ6 "starvation")).ti**) AND (exp *"Artificial Respiration"/ OR "mechanical ventilation".ti OR "mechanical ventilations".ti OR "Artificial Respiration".ti OR "Artificial Respirations".ti OR "High-Frequency Ventilation".ti OR "High-Frequency Jet Ventilation".ti OR "Interactive Ventilatory Support".ti OR "Liquid Ventilation".ti OR "Noninvasive Ventilation".ti OR "One-Lung Ventilation".ti OR "Positive-Pressure Respiration".ti OR "Continuous Positive Airway Pressure".ti OR "Intermittent Positive-Pressure Breathing".ti OR "Intermittent Positive-Pressure Ventilation".ti OR "Ventilator Weaning".ti OR *"mechanical ventilator"/ OR "mechanical ventilator".ti OR "mechanical ventilator".ti OR "mechanically ventilated".ti OR "artificially respirated".ti OR "artificially ventilated".ti **OR "pressure-support".ti OR "pressure-supported".ti OR "pressure-support*".ti**) NOT (exp "Animals"/ NOT exp "Humans"/) AND (exp "Treatment Outcome"/ OR "Treatment Outcome".mp OR "Outcome".mp OR "Outcomes".mp OR "Clinical Effectiveness".mp OR "Clinical Efficacy".mp OR "Treatment Effectiveness".mp OR "Treatment Efficacy".mp OR "Treatment Failure".mp OR exp "Intensive Care Unit"/ OR "Intensive Care".af OR "ICU".af OR exp "Intensive Care"/ OR "Critical Care".af OR "Critical Illness"/ OR "critical illness".af OR "critically ill".af) **NOT (exp *"Noninvasive Ventilation"/ OR exp *"Liquid Ventilation"/ OR exp *"High Frequency Jet Ventilation"/ OR exp *"One-Lung Ventilation"/ OR "Non-invasive Ventilation".ti OR "Noninvasive Ventilation".ti OR "Liquid Ventilation".ti OR "High-Frequency Jet Ventilation".ti OR "One-Lung Ventilation".ti OR "Non-invasive Ventilat*".ti OR "Noninvasive Ventilat*".ti OR "Liquid Ventilat*".ti OR "High-Frequency Jet Ventilat*".ti OR "One-Lung Ventilat*".ti OR "Non-invasive Respirat*".ti OR "Noninvasive Respirat*".ti OR "High-Frequency Jet Respirat*".ti OR "One-Lung Respirat*".ti)**)
